# The Impact of Deviation of the Stun Shot from the Ideal Point on Motor Paralysis in Cattle

**DOI:** 10.3390/ani10020280

**Published:** 2020-02-11

**Authors:** Vladimir Vecerek, Josef Kamenik, Eva Voslarova, Martina Volfova, Zuzana Machovcova, Jarmila Konvalinova, Lenka Vecerkova

**Affiliations:** 1 Department of Animal Protection and Welfare and Veterinary Public Health, Faculty of Veterinary Hygiene and Ecology, University of Veterinary and Pharmaceutical Sciences Brno, 612 42 Brno, Czech Republic; vecerekv@vfu.cz (V.V.); volfovam@vfu.cz (M.V.); machovcovaz@vfu.cz (Z.M.); konvalinovaj@vfu.cz (J.K.); V13120@vfu.cz (L.V.); 2 Department of Food Hygiene and Technology and Gastronomy, Faculty of Veterinary Hygiene and Ecology, University of Veterinary and Pharmaceutical Sciences Brno, 612 42 Brno, Czech Republic; kamenikj@vfu.cz

**Keywords:** stunning, shot position, collapse, animal welfare

## Abstract

**Simple Summary:**

The welfare of cattle during slaughter predominantly depends on the ability of the stunner to induce immediate collapse and insensibility. The main aim of the study was to evaluate the accuracy of the stun shot under commercial conditions and the success rate of inducing motor paralysis in relation to the distance of the placement of the stun shot from the ideal point on the cattle skull. The results show that as the deviation in the stun shot position increases, the failure to induce motor paralysis in cattle significantly (*p* < 0.01) increases.

**Abstract:**

The effect on motor paralysis of a deviation in the stun shot placement from the ideal point on cattle skulls was monitored in 627 bovine animals (271 bulls and 356 cows) stunned with a captive bolt during slaughter in a slaughterhouse. The number of animals that experienced motor paralysis and the necessary fall of the animal in the stunning box were recorded after the stun shot. Subsequently, the position of the stun shot was measured on the skull of the slaughtered cattle in relation to the ideal point on the skull, and at a deviation from the ideal point, the quadrant on the skull in which the bullet was located was determined. The results show that with the increasing distance of the placement of the stun shot from the ideal point on the skull, the incidence of failure to induce motor paralysis in cattle increases significantly (*p* < 0.01) from 2.4% (within 3 cm of deviation) to 72.2% (at deviations > 7 cm). There was a significant increase in the failure to induce motor paralysis in bulls as well as in cows, but this was more frequent in bulls regardless of the magnitude of the deviation from the ideal point (with the exception of a distance greater than 7 cm where the chances of inducing motor paralysis in bulls and cows are equally low). The incidence of failure to induce motor paralysis in cattle was not dependent on the placement of a stun shot in various quadrants on the skull. With the increasing deviation in any direction from the ideal point, the likelihood of effective stunning of cattle decreases. The results are important from the animal welfare point of view of the slaughter of cattle, and demonstrate the necessity of optimum placement of the stunning shot on the bovine skull in order to achieve the successful motor paralysis of cattle during their stunning at the slaughterhouse.

## 1. Introduction

For welfare reasons, cattle have to be stunned before bleeding at slaughter. The objective is to immediately induce a deep unconsciousness without causing any pain [1]. Currently, penetrative captive bolts are the most frequent stunning tools used in cattle slaughtering [2,3]. This method of stunning damages the brain structures that are engaged in the state of consciousness. Nevertheless, brain damage caused by captive bolts is dependent on a number of effects: the shock wave induced in the brain when the bolt hits the skull, mechanical destruction when the bolt penetrates the brain, and the level of damage to the brain tissue when the bolt is pulled out of the wound, during which bleeding occurs as a result of structural changes. The location of the shot has a great influence on all of these aspects [1]. In the case of stunning with a captive bolt device, this device should be placed on the frontal region of the head in such a way that the bolt impacts the brain stem [4]. Even if the brain stem is not physically hit, the shot causes cerebrum destruction and from there the transferred kinetic energy and the increased pressure inside the skull are transmitted to the brain stem. Consequently, the shock wave causes neuronal depolarization. In addition, the resulting massive bleeding also affects the brain stem.

A correctly functioning brain stem and thalamus are essential for keeping consciousness, and damage to either of these areas causes a fast loss of consciousness [3]. On the contrary, failure to damage the brain stem leaves the animal conscious and sensitive to intolerable pain. Under field conditions, determining and hitting the ideal point and direction of a stun shot can be difficult and deviations occur. The accuracy of shooting during cattle slaughter in two cattle head deboning plants was examined by Fries et al. [4] and they found that the position of shots varied significantly. In 64.7% of cases in the first plant and 65.3% in the second plant, the skull shots were within a range of a maximum distance of 0 to 2.5 cm from the ideal position. Medium precision (shots within a range of a maximum distance of 3 to 4.5 cm) was recorded in 31.3% and 31.5% of cases with 4.0% and 3.1%, respectively, of the skull shots showed poor accuracy (shots ranging > 5 cm from the ideal position). Gregory et al. [5] investigated the shot position on the bovine skull at an abattoir. In this case, if the distance from the ideal shooting position exceeded 2 cm, the shot position was considered incorrect or inaccurate. For all cattle, 48.7% of shots were within a 2 cm range of the ideal position. In 43.5% of animals, a higher head shot that was over 2 cm above the ideal shooting position was found. Bulls had a lower probability than other cattle of getting a high head shot (36% vs. 45% of high shots). Wider or lower skull shots were registered in 7.7% and 0.1% of animals, respectively. Atkinson et al. [6] assessed shot precision in cattle slaughtered under commercial conditions. They found inaccurate shooting (projectile hits located more than 2 cm outside of the ideal position) in 8.0% of all cattle (8.5% of bulls, 7.1% of cows, 3.4% of steers and 14.0% of calves), with 6% of cattle being shot above, 1.8% being shot beside and 0.2% below. Von Wenzlawowicz et al. [7] carried out 50 assessments in 25 cattle abattoirs during routine slaughter procedures and they found great differences in shot accuracy, especially in relation to the types of stunning pens, slaughtering speed and the use of pneumatic as opposed to cartridge-fired stunners. A deviation in the placement of the stunning shot may result in imperfect stunning. Therefore, after stunning, it is essential to confirm that the animal is insensitive to pain and in a permanent state of unconsciousness before it is hoisted onto the bleeding line [8]. An immediate and permanent loss of posture is considered a reliable indicator of unconsciousness [5,9].

The aims of this study were to find out whether the failure to induce motor paralysis in cattle after a stunning shot by a captive bolt depends on the distance of the stunning projectile from the ideal point, and on the quadrant where the stunning shot is placed on the cattle skull; the differences between bulls and cows were also assessed.

## 2. Materials and Methods

The monitoring was carried out during routine slaughter at the two largest bovine slaughterhouses in the Czech Republic. All cattle stunned and killed in two days were investigated at each plant. In total, 627 animals were examined: 271 bulls (aged between 12 and 92 months, average weight of 735.6 ± 8.4 kg) and 356 cows including heifers (aged between 13 and 210 months, average weight of 505.5 ± 7.9 kg).

Stunning was performed in a conventional stunning box without mechanical head or body fixation. After the animal’s head was placed in a suitable position, it was shot with a Matador SS 3000 B trigger-activated captive-bolt gun (Termet, France) using caliber 6.3/12 red explosive cartridges (powder content in the cartridge was 320 mg) intended for all animals over 450 kg (beef, bulls, young cattle, cows, heifers). Two guns were available and were used alternately for each shooting to limit heating (a gun did not exceed a firing rate of less than 2 min). The slaughter rate was approximately 40 animals per hour.

All animals were shot by certified slaughtermen who had received the appropriate education as required by Czech legislation. Two observers were standing behind the stunning pen and recorded the identification number of each animal. After the captive bolt was fired, they recorded for each animal whether or not there was motor paralysis and the necessary fall of the animal in the stunning box. Animals that failed to collapse were re-shot. For the purposes of the study, only the position of the first shot was considered. When more shots were fired, notes were made on the position and the order of shot holes on the head at the time of stunning to identify the first shot during subsequent measurement.

**Figure 1:**
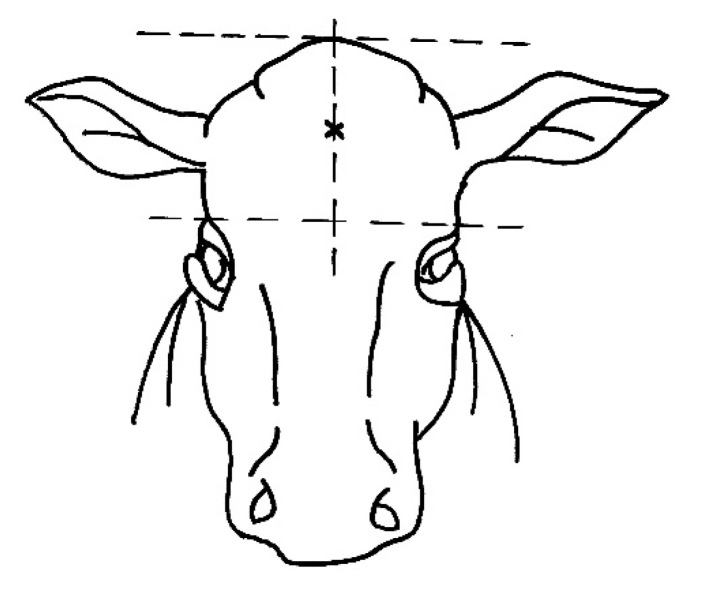
The cross indicates the ideal location of the stun shot.

**Figure 2:**
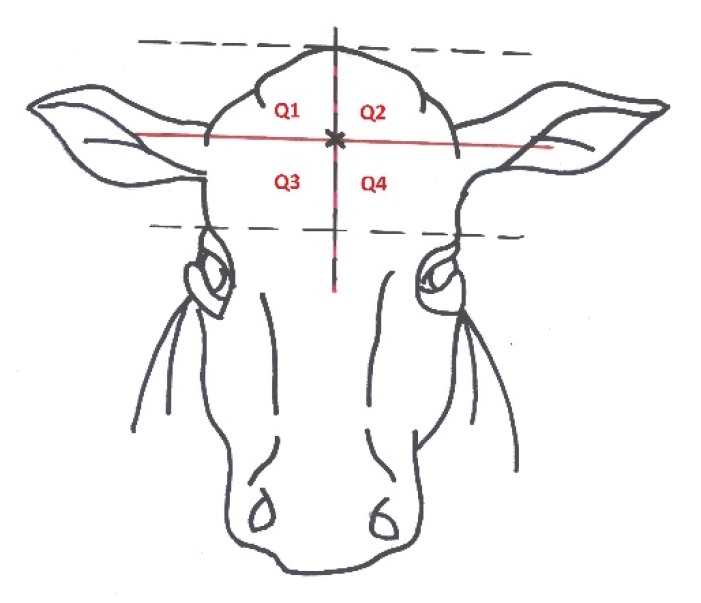
Division of quadrants on the head of cattle.

The skull of each animal assessed at stunning was inspected after decapitation and skinning and the shot location was recorded and categorized on the basis of its deviation from the ideal point (0 to 3 cm, 3.1 to 5 cm, 5.1 to 7 cm, > 7 cm). The center of the line segment placed vertically between two horizontal lines (a vertical line segment is perpendicular to both horizontal lines) is considered to be an ideal point for the stunning shot in bulls and cows. The first line goes horizontally as the eye line. The second horizontal line passes through the highest point of the skull and is parallel to the first horizontal line (Figure 1). In the case of a deviation in the location of the stun shot from the ideal point, the quadrant in which the bullet was located was determined, looking at the skull from the front quadrant 1 = upper left, quadrant 2 = upper right, quadrant 3 = lower left, quadrant 4 = lower right (Figure 2).

The frequency of unsuccessful induction of motor paralysis was assessed according to the distance of the stun shot from the ideal point on the skull and the quadrant in which the stun projectile was positioned. The occurrence of failed motor paralysis was determined for total cattle, and separately for bulls and cows.

The statistical package Unistat v. 6.5. (Unistat Ltd., London, England) was used for the analysis of the data. The Chi-square test (with Yates correction) within the 2 × 2 contingency table procedure was used to perform the statistical comparisons between the frequencies of the categorical variables of interest. In the case where the frequencies in the contingency table were under 5, a Fisher exact test was used instead of the Chi-square test [10]. A *p*-value of 0.05 was considered significant. To determine the relationship between the occurrence of unsuccessful induction of motor paralysis and the distance of the stun shot from the ideal point on the skull, a regression analysis in Excel Microsoft Office Professional Plus 2013 was performed.

## 3. Results

A total of 271 bulls and 356 cows were slaughtered, of which 44 bulls and 16 cows did not collapse after the stun shot. The overall rate of failure to induce motor paralysis after the stun shot was 9.6%. The number of animals slaughtered and the number of animals that did not undergo motor paralysis after the stun shot being placed at different distances from the ideal point on the bovine skull are given in Table 1.

**Figure 3:**
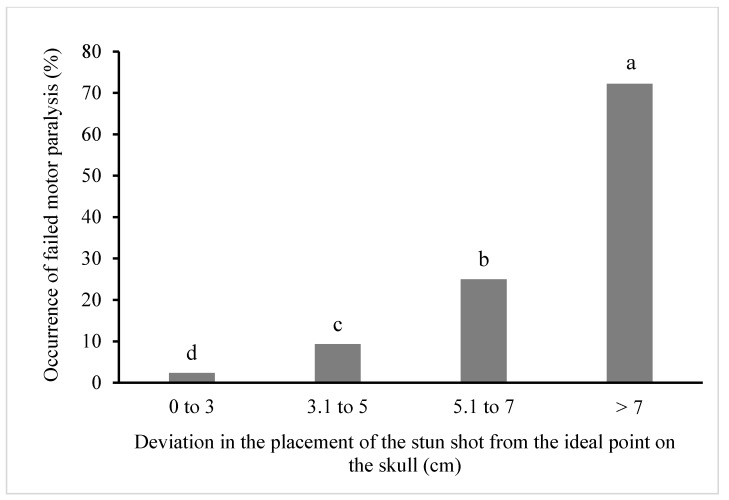
Percentage of unsuccessful motor paralysis in cattle in relation to the distance of the stun shot from the ideal stunning point. ^a–d^ Columns with different superscripts differ significantly (*p* < 0.01).

The influence of the stun shot distance from the ideal point on the skull on the failure to induce motor paralysis in cattle is shown in Figure 3. The results show that with the increasing distance of the stun shot placement from the ideal point on the bovine skull, the incidence of failure to induce motor paralysis in cattle increases from 2.4% (at deviations up to 3 cm) to 72.2% (at deviations exceeding 7 cm). The frequency of unsuccessful induction of motor paralysis differed (*p* < 0.01) between all classes of stun shot placement deviations. The relationship between the occurrence of unsuccessful induction of motor paralysis and the distance of the stun shot from the ideal point on the skull showed a quadratic dependence expressed by the function y = 1.5290x^2^ − 6.481x + 7.1883 with the value R^2^ = 0.9584.

**Figure 4:**
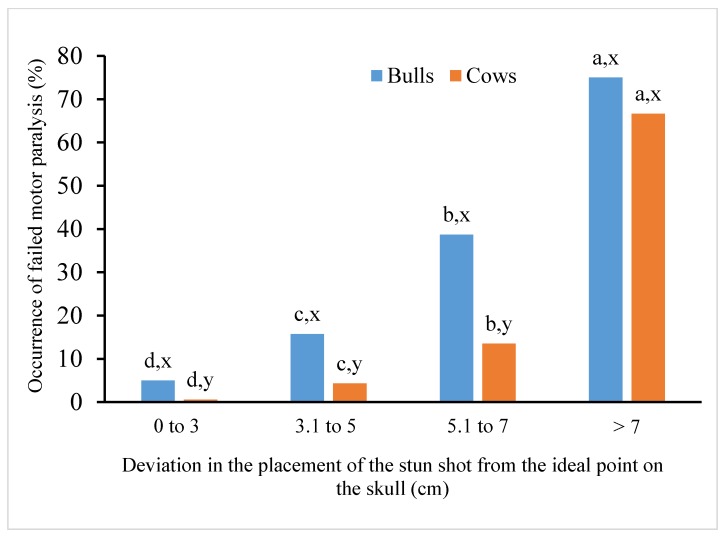
Percentage of unsuccessful motor paralysis in bulls and cows depending on the distance of the stunning shot from the ideal stunning point. ^a–d^ Columns within the same category of cattle with different superscripts differ significantly between classes of deviation (*p* < 0.05); ^x,y^ columns within the same deviation class with different superscripts differ significantly between cattle categories (*p* < 0.05).

The influence of the distance of the stun shot placement from the ideal point on the bovine skull on the failure to induce motor paralysis in bulls and cows is shown in Figure 4. The results show that with the increasing distance of the stun shot placement from the ideal point on the skull, the percentage of failure to induce motor paralysis increases in both bulls and cows.

There was an increase in the failure rate in bulls from 5.0% (deviations up to 3 cm) to 75.0% (at deviations exceeding 7 cm). The relationship between the occurrence of unsuccessful induction of motor paralysis in bulls and the distance of the stun shot from the ideal point on the skull showed a quadratic dependence expressed by the function y = 1.9120x^2^ + 0.1789x + 1.2021 with the value R^2^ = 0.8892.

In cows, the increase was from 0.6% (deviations up to 3 cm) to 66.7% (at deviations exceeding 7 cm). The relationship between the occurrence of unsuccessful induction of motor paralysis in cows and the distance of the stun shot from the ideal point on the skull showed a quadratic dependence expressed by the function y = 2.3056x^2^ − 13.654x + 16.055 with the value R^2^ = 0.7452.

The percentage of unsuccessful induction of motor paralysis differed (*p* < 0.05) between all classes of deviations in the stun shot placement in bulls as well as cows. Statistically significant differences (*p* < 0.05) were also found when comparing the percentage of failure to induce motor paralysis in bulls and cows for all classes of the stun shot deviation from the ideal point on the bovine skull, except for distances greater than 7 cm.

**Figure 5:**
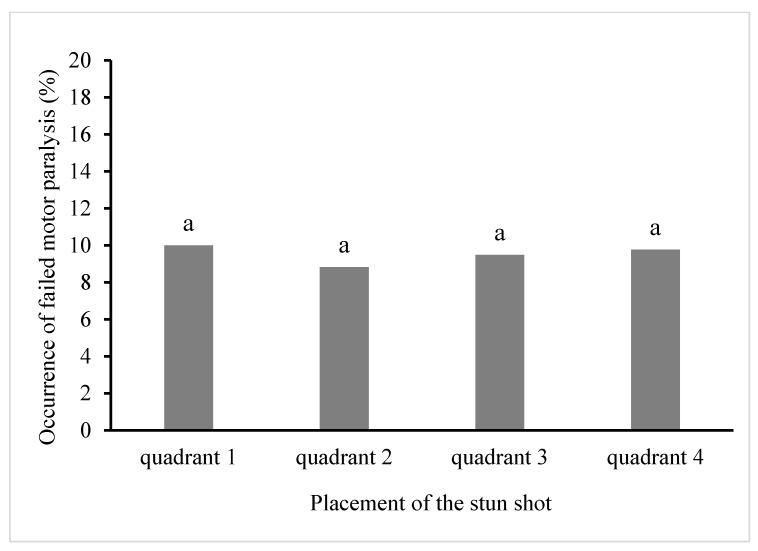
Percentage of unsuccessful motor paralysis in cattle, depending on the location of the stun shot in the skull quadrant. ^a^ Columns with the same superscript do not differ (*p* > 0.05).

The absolute frequencies of the stun shot placement in a different quadrant of a bovine skull and the number of animals that did not collapse after the first stun shot placed in a different quadrant of the skull are given in Table 2. The percentage of non-induction of motor paralysis in cattle depending on the location of the stunning shot in a particular quadrant of the skull is shown in Figure 5. The results show that the rate of failure to induce motor paralysis in cattle does not change significantly (*p* > 0.05) depending on the quadrant of the stunning shot placement on the skull.

**Figure 6:**
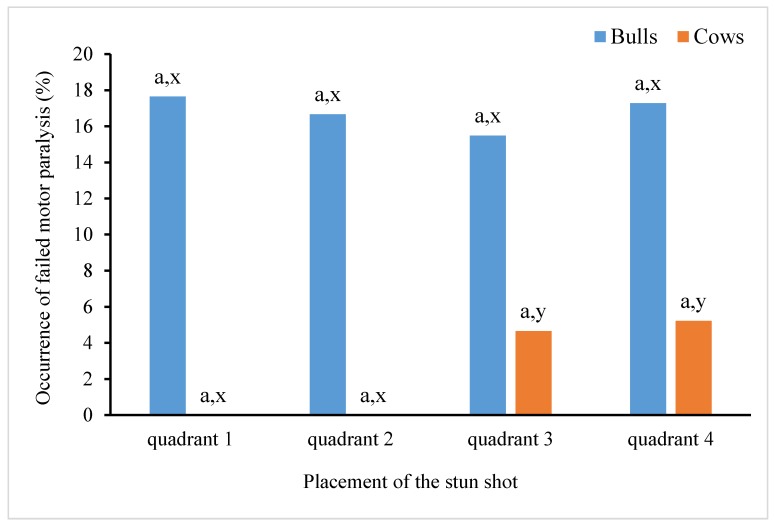
Percentage of unsuccessful motor paralysis in bulls and cows depending on the quadrant of the skull where the stun shot was located. ^a^ Columns within the same cattle category with the same superscript do not differ between quadrants (*p* > 0.05); ^x,y^ columns within the same quadrant with different superscripts differ significantly between cattle categories (*p* < 0.01).

Figure 6 shows a comparison of the percentage of non-induction of motor paralysis in bulls and cows depending on the skull quadrant in which the stun shot was placed. For both bulls and cows there was no difference (*p* > 0.05) in the occurrence of unsuccessful induction of motor paralysis when the stun shots were placed in different quadrants. However, non-induction of motor paralysis was more frequent (*p* < 0.01) in bulls than in cows when the stun shots were placed in quadrants 3 and 4.

## 4. Discussion

The results show considerable variability in the placement of the stun shot under routine slaughterhouse conditions. Only less than half (46.9%) of the animals had a wound on the skull within 3 cm of the ideal point, while the others were shot with a greater deviation in the distance. In 18 animals (2.9%), the shot on the skull was more than 7 cm from the ideal point. The lack of shooting accuracy during cattle slaughter has also been pointed out by other authors [4,5,6,7]. The lack of precision may be caused by a lack of competence or by poor supervision or work pressure from the lairage [4]. According to Von Wenzlawowicz et al. [7], high slaughter speed (>30 animals per hour) may result in lower shooting accuracy as the slaughtermen have less time. This may be the case in the slaughterhouses monitored in our study, where approximately 40 animals per hour were slaughtered.

Another reason for inaccurately aimed shots might be wrong positioning or missing restraint of the head when a sudden movement of the head during stunning can occur [4]. According to Von Holleben [11], if plants exceed a certain slaughter speed, additional restraining equipment should be installed within the stunning pen to achieve better shooting accuracy. Council Regulation (EC) No 1099/2009 on the protection of animals at the time of killing requires restraining boxes for cattle used in connection with a pneumatic captive bolt to be fitted with a device that prevents both the lateral and vertical movement of the head [12].

However, in the case of cartridge-fired stunners, no specific requirements for the restraint of the head are prescribed by law and therefore their use in practice varies. However, from the practical impact point of view, it is essential to avoid sudden head movement leading to a deviation in the stun shot placement especially in case of using cartridge-fired stunners. According to Gregory [5] and Von Wenzlawowicz et al. [7], shooting accuracy becomes less crucial if high-powered devices are used. However, as shown by the results of our study assessing cartridge-fired stunners, the deviation in the stun shot placement on the head is conclusively associated with a lower stunning success rate. With the increase in the distance of the wound on the skull from the ideal point, the incidence of failure to induce motor paralysis in cattle increased from 2.4% (at the deviations up to 3 cm) to 72.2% (at the deviations > 7 cm).

On the other hand, even placing the stunning shot in close proximity to a point considered by many authors as ideal did not guarantee 100% success in inducing immediate and permanent loss of posture. According to Daly et al. [13], there is a significant variation in the severity of effects on brain function despite careful, accurate positioning of the stunner. Conversely, even in spite of a deviation of more than 7 cm in the stun shot position, some animals were stunned properly. The efficiency of stunning is not only dependent on the placement of the stunner or on the projectile direction. Under certain circumstances, even a shot that was not in the ideal position or not at the ideal angle could induce unconsciousness as a consequence of concussion to the brain. Nevertheless, according to the anatomy of the skull, the probability of good efficiency is higher if the gun is located at the center of the forehead, since there is a higher likelihood that the bolt will penetrate and impair the brain stem [4].

The incidence of unsuccessful stunning increased in both bulls and cows as the distance from the stun shot to the ideal point on the skull increased. However, in cows, the success rate for inducing motor paralysis after stunning was higher than in bulls regardless of the shot placement. The only exceptions were wounds placed more than 7 cm from the ideal place, where the chances of successful stunning in bulls and cows were equally low. Similarly, Atkinson et al. [6] found that bulls were three times more likely to be inadequately stunned compared with other cattle categories. Gregory et al. [5] reported that 4.2% of bulls and 2.3% of heifers failed to collapse after the first shot.

In contrast, Gouveia et al. [14] reported that females have a higher proportion of unsuccessful stuns in comparison with males. However, the authors explain this finding by the higher percentage of females over 30 months of age compared to males in their dataset. In animals older than one year the occurrence of improper stuns corresponded with our results, i.e., a higher incidence in males than females. Gouveia et al. [14] suggest that the differences in skull structure between the sexes may become established only after the first year of the animals’ life, increasing the difficulty of a successful stun in bulls. As there were no animals under one year of age monitored in our study, we cannot confirm this finding. The age of cattle slaughtered at abattoirs during our observation ranged from 12 to 92 months in bulls and from 13 to 210 months in cows (including heifers). However, it can be expected that since adult cattle have a larger and thicker frontal bone, in some animals the brain might be beyond the normal reach of the bolt [14]. Correspondingly, older bulls tend to have a thicker bone mass on the forehead than other cattle categories, thus a higher resistance to the kinetic energy delivered by the shot. This could be an essential contributing factor to the lower efficacy of stunning in bulls compared to other bovine classes [6].

An analysis of the direction in which the deviation of the stun shot from the ideal point occurred revealed that the shots placed below the ideal point, both to the left (55.5%) and right (34.3%), were significantly more frequent. In low-placed hits, motor paralysis failed to be induced more often in bulls than in cows. Wounds placed above the ideal point level were detected in only 64 heads of cattle (10.2%). On the contrary, Gregory et al. [5] and Atkinson et al. [6] found fewer head shots below the ideal position in comparison with high and wide head shots. In terms of the success of inducing motor paralysis in relation to the placement of the stun shot in different quadrants of the skull; however, we did not find any difference between animals shot in different quadrants. If the placement is too high, the bolt will only reach the cerebellum, if it is too low, the frontal cortex will be damaged only superficially. In both cases, the animal is wounded but conscious [15].

## 5. Conclusions

With the increasing deviation in any direction from the ideal point, the likelihood of effective stunning of cattle decreases. The results are important from the perspective of cattle welfare and labor safety during slaughter, and demonstrate the necessity of optimal placement of the stun shot on the bovine skull in order to achieve successful motor paralysis and thus stunning of cattle at the slaughterhouse.

## Figures and Tables

**Table 1:** Number of animals slaughtered and that failed to collapse in relation to the distance from the location of the stun shot from the ideal point.

	Distance of the Stun Shot from the Ideal Point (cm)			
	0 to 3	3.1 to 5	5.1 to 7	>7
**Number of bulls slaughtered**	120	108	31	12
**Number of bulls that failed to collapse after the stun shot**	6	17	12	9
**Number of cows slaughtered**	174	139	37	6
**Number of cows that failed to collapse after the stun shot**	1	6	5	4
**Total number of cattle slaughtered**	294	247	68	18
**Total number of cattle that failed to collapse after the stun shot**	7	23	17	13

**Table 2:** Number of animals slaughtered and that failed to collapse after a stun shot placed in a different quadrant of the skull.

	Placement of Stun Shot			
	Quadrant 1	Quadrant 2	Quadrant 3	Quadrant 4
**Number of bulls slaughtered**	17	18	155	81
**Number of bulls that failed to collapse after the stun shot**	3	3	24	14
**Number of cows slaughtered**	13	16	193	134
**Number of cows that failed to collapse after the stun shot**	0	0	9	7
**Total number of cattle slaughtered**	30	34	348	215
**Total number of cattle that failed to collapse after the stun shot**	3	3	33	21
